# Empathy and Its Predictive Factors in Undergraduate Health Professional Students: A Longitudinal Cohort Study

**DOI:** 10.3390/nursrep15090316

**Published:** 2025-08-28

**Authors:** Valeria Caponnetto, Elona Gaxhja, Ilda Taka, Elona Prifti, Vittorio Masotta, Ilaria Paoli, Loreto Lancia, Angelo Dante, Cristina Petrucci

**Affiliations:** 1 Department of Life, Health and Environmental Sciences, University of L’Aquila, 67100 L’Aquila, Italy; valeria.caponnetto@univaq.it (V.C.); vittorio.masotta1@univaq.it (V.M.); ilaria.paoli@graduate.univaq.it (I.P.); loreto.lancia@univaq.it (L.L.); cristina.petrucci@univaq.it (C.P.); 2 Nursing Department, University “Aleksander Xhuvani” of Elbasan, 3001 Elbasan, Albania; elona.gaxhja@uniel.edu.al; 3 Department of Technical-Medical Specialties, University “Aleksander Xhuvani” of Elbasan, 3001 Elbasan, Albania; ilda.taka@uniel.edu.al; 4 Clinical Department, University “Aleksander Xhuvani” of Elbasan, 3001 Elbasan, Albania; elona.zhiva@uniel.edu.al

**Keywords:** healthcare students, empathy, predictors, educational strategies

## Abstract

**Background:** Empathy is essential for enhancing care quality, making its understanding and predictors crucial for healthcare education. **Objective:** To investigate empathy evolution and its predictors among first-year health professional students at a university in Albania. **Methods:** A longitudinal cohort study was conducted on a total of 206 participants (78.2% female, mean age 18.4 years), with empathy assessed at baseline and program completion using the Jefferson Scale of Empathy-Health Professional Students. **Results**: The findings revealed stable empathy levels overall (*p* = 0.369), with no significant differences between nursing and other students. Approximately 52.9% experienced empathy gains, while 44.7% experienced losses, yielding an average score change of +0.7 (SD = 14.9). A younger age and lower baseline empathy scores were significant predictors of empathy gains, as shown by regression analyses. **Conclusions:** The study highlights a dual empathy trajectory among students and emphasizes a person-centered approach to health professional education to foster empathy development.

## 1. Introduction

Semantically, empathy is defined as “the action of understanding, being aware of, being sensitive to, and vicariously experiencing the feelings, thoughts, and experience of another of either the past or present without having the feelings, thoughts, and experience fully communicated in an objectively explicit manner” [1].

In healthcare education, empathy is recognized as a cognitive and affective feature of students that primarily involves understanding patients, being aware of this understanding, and having the motivation to alleviate patients’ suffering [2]. This definition highlights the crucial role of empathy in improving the quality of care, since it ensures a better student–patient relationship, effective communication, and proactive care [3,4,5,6]. Knowing the level of empathy, its evolution, and the factors that influence this evolution in students helps educators adopt the best teaching strategies to improve this essential soft skill in students [7,8,9,10].

Available evidence shows that the empathy levels of healthcare students may differ according to some individual factors, such as gender, self-efficacy, and self-esteem, or the healthcare program they are enrolled in and the time spent in the program [11,12,13]. Notably, the literature reports inconsistent results regarding the empathy levels of nursing students, who showed both significantly higher and lower levels when compared to their peers enrolled in other health-related programs [13,14].

The evolution of empathy over time seems also to vary according to other factors, such as the adopted training strategies and teaching models, the progression of students in their course of study, and the study workload [9,15,16,17,18,19,20]. However, considering intra-individual variability, it is important to note that even if students attend the same course, their empathic evolution can differ over time. In fact, some students can achieve an empathy gain, while their course mates can experience a loss or no change [8,15,17]. Despite its relevance, this intra-individual perspective is rarely adopted in existing research. Capturing individual trajectories of empathy change provides a more nuanced and dynamic understanding of empathy development, offering valuable insights for tailoring educational interventions to students’ evolving needs. These insights also lead to the hypothesis that individual characteristics may explain the different trajectories of students’ empathy throughout a program. The predictive power of factors that influence empathy evolution in healthcare students seems to vary across countries and cultures [21,22,23]. Among the variables hypothesized to affect empathy development, pet ownership has gained attention, as evidence suggests that interactions with animals may positively contribute to the cultivation of empathetic abilities [24,25].

Given the inconsistencies reported in the literature regarding the empathy levels of nursing students, this study aimed to investigate the differences in empathy evolution over time between nursing and other health professional students, as well as the role of individual variables [13,23] in predicting these changes. The study was conducted in Albania, where this phenomenon has not yet been explored.

The study hypotheses were (a) nursing students and other health professional students have different levels of empathy both at the beginning and end of their programs; (b) different significant changes in the empathy levels of students occur according to the academic program attended; and (c) students’ characteristics can predict changes in empathy levels.

## 2. Materials and Methods

### 2.1. Study Design and Setting

A longitudinal cohort study was conducted from October 2017 (academic year 2017–2018) to June 2020 (academic year 2019–2020) at the University “Aleksander Xhuvani” of Elbasan, and its reporting was performed according to the STROBE guidelines [26]. The University of Elbasan is the second largest university in Albania. The university offers undergraduate health professional programs in Nursing, Midwifery, Physiotherapy, Speech Therapy, Imaging Technology, and Biomedical Laboratory Technology. To be admitted in one of these programs, students are selected according to their high school grades. Only students with a mean high school grade equal or above 6.5/10.0 can apply to these programs. In addition, during high school, candidates can take optional exams that align with their university choices. Students’ high school grades and grades in the optional exams are combined by a centralized institution called the Educational Services Center to provide a total ranking for each student. A candidate is admitted to a university program according to their ranking and preferences when applying. Each program lasts 3 years and requires students to obtain 180 university formative credits, equivalent to 4500 h of academic education. Students undergo theoretical lessons, practical workshops, seminars, and clinical placement. There are final exams for both theoretical courses and clinical placements. At the end of the program, students must pass a written examination and discuss a final dissertation to graduate [27]. Graduation sessions at the University of Elbasan are scheduled in July or September each year.

### 2.2. Participants

All students enrolled in one of the six health professional courses offered by the university in their first academic year in October 2017 were considered eligible for the study and invited to participate. Only students who gave their informed consent were enrolled in the study. At the beginning of each academic year, approximately 550 health professional students are admitted to the university. G* Power 3.1.9.2 software was used to estimate the sample size. A total of at least 172 students (86 from nursing and 86 from other programs) were required to provide a 90% power (1 − *β*) and 5% α error in detecting mean differences in empathy levels between the two groups of students [28]. The effect size (mean difference) was expected to be medium (0.5) [29].

### 2.3. Data Collection, Variables, and Risk of Bias

Data were collected at the beginning of the first year (T0 = October 2017) and at the end of the third year, just before graduation (T1 = June 2020).

At T0, the collected data were course of study, gender, age, entry ranking, has a pet (yes/no), has volunteered in a healthcare field in the last year (yes/no), reason for choosing the course (work opportunities/sheer interest), and empathy level. At T1, only the empathy level was measured.

Data were collected by paper and pencil, using an ad hoc questionnaire for participants’ characteristics and the Albanian version of the Jefferson Scale of Empathy-Health Professional Students (JSE-HPS) for empathy levels. The validated Albanian version of the JSE-HPS was valid and reliable for the measurement of the empathy levels of Albanian students [13]. The JSE-HPS is a 20-item tool based on a 7-point Likert scale that ranges from 1 (strongly disagree) to 7 (strongly agree). Empathy scores were computed according to the provided user guide and range between 20 and 140, with higher scores indicating higher empathic levels. Permission to use the JSE-HPS was obtained from the Center for Research in Medical Education and Health Care of the Jefferson Medical College of Thomas Jefferson University, USA.

Data were collected, both at T0 and T1, in a dedicated room. At T0, students were yet to officially begin lectures, while at T1, students had completed the educational pathway and were waiting for the graduation procedures. All data were then inserted into an electronic spreadsheet in Microsoft Excel (Microsoft 365, version 2507 build 19029.20208)^®^.

Some strategies were applied to minimize the risk of bias. Specifically, to reduce the risk of selection bias, all first-year students were invited to participate in the study, while the risk of detection bias was reduced by using a valid and reliable tool and maintaining the same conditions and modalities of data collection for all participants [30,31,32].

### 2.4. Ethics

The study complied with the Declaration of Helsinki. It was approved by the Internal Committee of the University “Aleksander Xhuvani” of Elbasan, Albania (No. 1883/2018). Participation in the study was voluntary. Students were informed that participation or non-participation in the study would not influence their academic pathway. Those who agreed to participate signed a written informed consent.

### 2.5. Statistical Analyses

To manage dropouts, differences in the socio-demographic characteristics of students who completed the study and those who dropped out were explored using the *χ*^2^ test and Mann–Whitney *U* test, as appropriate. In accordance with the study aims, students were preliminarily split into two groups, i.e., nursing and other students. Descriptive analyses were used to report the characteristics of participants and highlight any differences in the two groups. Categorical variables were summarized using frequencies and percentages, while continuous variables were summarized using averages and standard deviations (±SD). Comparisons of student groups were performed using the Mann–Whitney *U* test and calculating Cohen’s *d* with a 95% CI to better understand the magnitude of these differences. The paired Wilcoxon test was used to detect any significant changes in empathy levels over time. For the purposes of this study, ‘empathy gain’ was defined as any positive change in JES-HPS score between T0 (baseline) and T1 (end of the program), ‘empathy loss’ as any negative change, and ‘no change’ as a difference equal to zero.

Categorial variables were compared using the Chi-square test (χ^2^). The reliability of the Albanian version of the JES-HPS was explored using Cronbach’s α coefficient.

Univariate linear regression analyses were performed to identify potential predictors of changes in empathy score. Both continuous variables (age, entry ranking, and JES-HPS entry scores) and categorical variables (gender, has a pet, has volunteered in a healthcare field in the last year, reason for choosing the program, and course of study) were considered as potential predictors. Predictive power was expressed as a linear regression coefficient (*β*) with 95% confidence interval (95% CI). Multivariate regression analysis was conducted using stepwise method to assess if predictors reporting a *p*-value < 0.05 in univariate analyses were independently related to changes in empathy, adjusting for all other independent variables.

Missing data related to age, entry ranking, and reason for choosing the program were handled using pairwise deletion. In this approach, all available data pairs were retained for each analysis, and cases with missing values were excluded only from analyses involving those specific variables [33]. This method allowed for the maximum use of available information without excluding participants entirely. In this study, missing data were minimal and their impact on the analyses was negligible.

All data were analyzed using IBM SPSS version 25.0 (IBM Corp., Armonk, New York, NY, USA), with the accepted level of statistical error ≤ 5%.

## 3. Results

### 3.1. Participants’ Flow and Characteristics

Out of the 495 enrolled students, 206 students completed the study. This means that there was a dropout rate of 58.4%, which was mainly due to organizational issues related to the COVID-19 pandemic. At baseline, there were no differences in the socio-demographic characteristics of students who completed the study and those who dropped out (Table A1—Appendix A).

As reported in Table 1, participants were mainly females (78.2%), had an average age of 18.4 (SD = 0.8) years (median = 18.0; IQR = 1.0), and showed an average entry ranking of 7.4 (SD = 0.9) points out of 10 (median = 7.0; IQR = 1.0). About 55.8% of the participants had a pet, and 36.4% had volunteered in the healthcare field in the last year. Most of the participants (93.7%) chose the program out of sheer interest.

Comparing the baseline characteristics of participants, nursing students showed a higher median entry ranking (*p* = 0.027) and more frequently had a pet (*p* = 0.004). No differences emerged for the other variables.

### 3.2. Empathy Levels of Nursing and Other Health Professional Students

The JES-HPS showed satisfactory Cronbach’s α values at both considered timepoints (α T0 = 0.721, α T1= 0.761), confirming good internal reliability of the tool.

When compared with other health professional students, nursing students showed a slightly higher average level of empathy at baseline (107.2, SD = 12.1 vs. 105.7, SD = 13.0) and at the end of the study (108.7, SD = 11.5 vs. 105.8, SD = 13.5). However, these differences were not significant. Effect sizes were small, with Cohen’s *d* = 0.119 (95% CI: −0.155 to 0.393) at T0 and *d* = 0.230 (95% CI: −0.044 to 0.504) at T1, indicating a slight but consistent difference in favor of nursing students (Table 2).

The average empathy levels of both nursing (T0 = 107.2, SD = 12.1 vs. T1 = 108.7, SD = 11.5) and health professional students (T0 = 105.7, SD = 13.0 vs. T1 = 105.8, SD = 13.5) increased over time, but not significantly.

**Figure 1:**
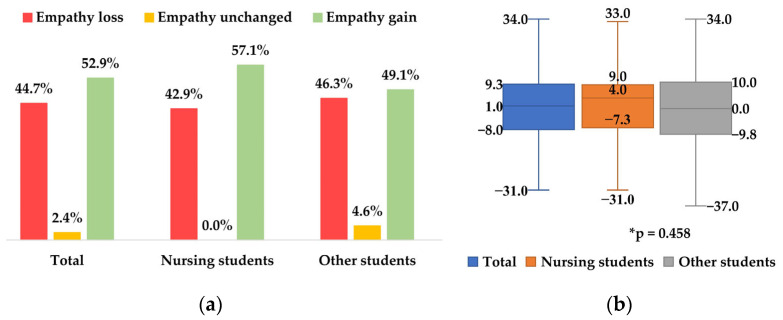
(**a**) Type of students’ empathy change. (**b**) Empathy score change in total sample and in nursing and other students (n = 206). * Mann–Whitney *U* test.

Beyond the absence of any significant changes in median empathy levels, the data revealed that at the end of their studies, 109 (52.9%) students achieved empathy gain, 92 (44.7%) demonstrated empathy loss, and 5 (2.4%) students had unchanged empathy scores, with similar figures for nursing and other health professional students (Figure 1a). The average change in JES-HPS scores was +0.7 (SD = 14.9), median = +1.0 (IQR = 17.3), with a high variability for the whole sample (minimum change = −31.0 and maximum change = +34.0); similar figures were detected for nursing and other health professional students, with no significant differences (Figure 1b). For this reason, the predictive analysis for change in empathy score was performed while merging the two groups of students into one sample. 

Univariate linear regression analysis, performed to identify the predictors of changes in empathy score, showed the predictive power of students’ age (β = −2.718, 95% CI = −5.266 to −0.171, *p* = 0.037) and JES-HPS score at T0 (β = −0.694, 95% CI = −0.826 to −0.562, *p* ≤ 0.001). Both variables confirmed their predictive power in the multiple linear regression analysis, with younger age (B = −2.849, 95% CI = −4.904 to −0.795, *p* = 0.007) and lower baseline empathy scores (B = −0.692, 95% CI = −0.822 to −0.562, *p* < 0.001) being significant predictors of an increase in empathy at the end of the program (Table 3).

Specifically, each one-year decrease in age at T0 was associated with a 2.85-point increase in the change in empathy score, and each one-point decrease JES-HPS score at T0 was associated with a 0.692-point increase. The model explained 36.7% of the variance in the change in empathy.

## 4. Discussion

Based on the study results and in contrast with the current literature [7,13,14], the hypothesis of a significant difference in empathy levels between nursing and other health professional students was rejected. In this study, nursing students reported a non-significantly higher average level of empathy than other health professional students. Despite the a priori power analysis guaranteeing an adequate sample size, caution should be exercised in generalizing the results of this study due to the high dropout rate. Even if a physiological dropout rate (range 5.7–35.5%) was expected due to student attrition [34], the disruption in education as a result of the COVID-19 pandemic was unforeseen. This could have reduced the chances of detecting significant differences in empathy levels between the two groups and over time. Hence, confirmative studies with larger sample sizes and lower participant dropout rates should be conducted. Also, the hypothesis of the positive impact of academic programs on the empathy levels of students was rejected. In this study, significant changes in empathy as a result of being enrolled in some programs, as documented by other authors [18,20,35], did not fully emerge. In this regard, focusing on the intra-individual empathy trajectory, almost all the students experienced changes in their empathy levels over time, with half of them achieving empathy gain while the others experienced empathy loss [17]. The academic programs considered allowed us to encounter the learning needs of only half of the students. Although empathy gain is a desired outcome, the finding that almost half of the students experienced empathy loss is equally important and deserves further consideration. As highlighted in the literature, empathy is a crucial determinant of care quality [3,4,5,6]. Therefore, a decline in empathy during the educational pathway raises concerns not only for individual development, but also for future clinical practice. This implies that when enrolling in nursing or other healthcare professional courses, students express different and hidden learning needs that should be coaxed out. In fact, some students may require teaching strategies aimed at improving their empathy levels, while others may benefit from strategies aimed at preventing any empathy loss. For this reason, to guarantee best educational results with direct implications for student–patient relationships, student-centered teaching approaches should be adopted [36,37,38]. Knowing the profile of students who achieve empathy gains and losses could be helpful for this purpose. In this study, younger and less empathetic students showed a greater increase in their empathy levels. These data provide educators with the knowledge that empathy could be more easily improved in younger students and that students’ empathy levels at enrolment cannot be ignored if a student-centered educational strategy is adopted. Over the years, different empathy training strategies have been documented in the literature. They can, start from specific activities, such as introducing new and specific activities [39,40,41,42,43], or even be a general re-arrangement of the educational pathway, but they must keep in mind that the final goal is to improve students’ empathy [16]. Although the described approaches were addressed to groups of students, they did not involve a student-centered perspective. A student-centered approach is suggested for future empathy training and research, considering the emerging insights into intra-individual evolution of empathy. Before new training strategies can be implemented, research should first extract the profile of students with a higher probability of empathy gain, investigating the power of individual, social, cultural, and environmental factors to predict changes in empathy. These data would ensure early identification of students with higher risks of empathy loss, so appropriate actions can be taken to modify some of the factors that determine empathy evolution. The strengths of this study include the strategies utilized to minimize selection and measurement biases, i.e., sample size estimation, involvement of all first-year students in all health professional programs at the university, and the use of a validated instrument to measure empathy (JES-HPS) [13].

### Limitations

This study has some limitations, one of which concerns the timing of the second data collection point (T1), which partially overlapped with the onset of the COVID-19 pandemic in Albania. The pandemic contributed to the high student dropout rate, suggesting that the study results should be generalized with caution, despite the ensured statistical power. During the first wave of the pandemic, Albanian students were withdrawn from clinical training, while theoretical lessons continued via online platforms. However, the longitudinal data collection reflects a broader temporal trajectory (T0: October 2017–T1: June 2020), suggesting that changes in empathy were influenced by multiple factors, including students’ academic progression, clinical experiences, and contextual events not limited to the pandemic, which occurred only at the end of the study period. Nonetheless, the pandemic may have affected students both directly, through emotional exposure to distressing situations (e.g., patient isolation, restrictive measures, unexpected deaths), and indirectly, by disrupting clinical training and increasing stress levels [44].

## 5. Conclusions

Considering the importance of empathy for patient outcomes and healthcare students’ education, this study longitudinally investigated the empathy levels of health professional students, highlighting the predictive role of baseline characteristics in empathy change. JES-HPS scores suggested no overall evolution in empathy throughout the program, with no significant differences between nursing and other health professional students. However, intra-individual analyses showed two opposing trajectories of change in students’ empathy, namely gain and loss, each occurring in almost half of the total sample. Change in empathy was predicted by younger age and lower baseline empathy levels. Therefore, students showed different educational needs regarding empathy when starting a healthcare professional program. In light of these findings, adopting a more student-centered perspective when evaluating empathy change and planning educational pathways is recommended. Furthermore, this study contributes to a deeper understanding of empathy as a dynamic and modifiable competence, reinforcing the importance of longitudinal monitoring at the individual level, focusing on within-student trajectories rather than relying solely on cohort averages. An early assessment of empathy level at program entry is recommended to identify students who may benefit from targeted strategies, with the dual aim of promoting empathy gains and preventing losses. The information obtained can guide the early implementation of an educational action with long-term benefits for patient care and professional practice. The finding that younger students and those with lower baseline empathy are more likely to experience empathy gains suggests that targeted educational support could directly enhance the quality of future healthcare provider–patient relationships. For this reason, further strengthening the embedding of empathy development within professional competency frameworks and accreditation requirements could help ensure that this competence is systematically nurtured throughout training, aligning nursing education with broader healthcare policy goals and reinforcing empathy as a recognized core element of nursing science. Additional research is needed to better characterize the profiles of students most likely to experience changes in empathy, thereby enabling the design and early implementation of strategies that could prevent loss of empathy by students and facilitate empathy gain.

## Figures and Tables

**Table 1:** Characteristics of participants at T0 (n = 206).

	Total Sample (n = 206)		Nursing Students (n = 98)		Other Students (n = 108)		*p*-Value
Gender, n (%)							
Male	45	21.8	23	51.1	22	48.9	0.591 *
Female	161	78.2	75	46.6	86	53.4	
Age, average (SD)							
Years	18.4	(0.8)	18.3	(0.7)	18.5	(0.8)	0.197 ^†^
Entry ranking, average (SD)							
Points/10	7.4	(0.9)	7.5	(0.9)	7.2	(0.9)	0.027 ^†^
Has a pet, n (%)							
No	91	44.2	33	36.3	58	63.7	0.004 *
Yes	115	55.8	65	56.5	50	43.5	
Has volunteered in a healthcare field in the last year, n (%)							
No	131	63.6	62	47.3	69	52.7	0.926 *
Yes	75	36.4	36	48.0	39	52.0	
Reason choice program, n (%)							
Sheer Interest	193	93.7	95	49.2	98	50.8	0.068 *
Work opportunities	13	6.3	3	23.1	10	76.9	

* χ^2^ test; ^†^ Mann–Whitney *U* test.

**Table 2:** JES–HPS scores of participants at different timepoints (n = 206).

Characteristics	Total Sample Average (SD)	Nursing Students Average (SD)	Other Students Average (SD)	*p*-Value	Cohen’s *d* [95% CI]
T0	106.4 (12.6)	107.2 (12.1)	105.7 (13.0)	0.544 ^†^	0.119 [−0.155 to 0.393]
T1	107.1 (12.7)	108.7 (11.5)	105.8 (13.5)	0.176 ^†^	0.230 [−0.044 to 0.504]
*p*-value	0.369 *	0.227 *	0.863 *	-	-

^†^ Mann–Whitney *U* test; * Wilcoxon paired test.

**Table 3:** Multiple linear regression analysis of predictors of change in empathy scores among health professional students.

Independent Variable	Unstandardized Coefficient (B)	Standard Error (SE)	Standardized Coefficient (β)	t	*p*-Value	95% CI for (B)
Age	−2.849	1.042	−0.154	−2.734	0.007	−4.904 to −0.795
JES-HPS (T0)	−0.692	0.066	−0.587	−10.463	<0.001	−0.822 to −0.562

Notes: R = 0.605; R^2^ = 0.367; Adjusted R^2^ = 0.360; F(2, 201) = 58.145; *p* < 0.001.

## Data Availability

The original contributions presented in this study are included in the article. Further inquiries can be directed to the corresponding authors.

## Public Involvement Statement

No public involvement in any aspect of this research.

## Guidelines and Standards Statement

This manuscript was drafted against the STROBE guidelines.

## Abbreviations

The following abbreviations are used in this manuscript:

STROBE	Strengthening the Reporting of Observational Studies in Epidemiology
JSE-HPS	Jefferson Scale of Empathy-Health Professional Students

## Appendix A

**Table A1:** Baseline characteristics of completing and dropout students.

Characteristics	Completing Students (n = 206)	Dropout Students (n = 289)	*p*-Value
Gender, n (%)			
Females	161 (42.0)	222 (58.0)	0.726 *
Males	45 (40.2)	67 (59.8)	
Age, average (SD) °			
Years	18.4 (0.8)	18.6 (1.3)	0.198 ^†^
Entry ranking, average (SD) °			
Points/10	7.4 (0.8)	7.3 (0.9)	0.264 ^†^
Has a pet, n (%)			
Yes	115 (43.2)	151 (56.8)	0.432 *
No	91 (39.7)	138 (60.3)	
Has volunteered in a healthcare field in the last year, n (%)			
Yes	75 (40.8)	109 (59.2)	0.767 *
No	131(42.1)	180 (57.9)	
Reason choice program, n (%) °			
Sheer Interest	193 (42.3)	263 (57.7)	0.394 *
Work opportunities	13 (35.1)	24 (64.9)	
Course			
Nursing	98 (56.0)	77 (44.0)	≤0.001
Other Courses	108 (33.8)	212 (66.2)	
JES–HPS score at T0, average (SD)	106.5 (12.6)	105.7 (12.9)	0.611 *

° missing data; * χ^2^ test; ^†^ Mann–Whitney U test.

## References

[1] Merriam-Webster. https://www.merriam-webster.com/dictionary/empathy.

[2] Hojat M., LaNoue M. (2014). Exploration and confirmation of the latent variable structure of the Jefferson scale of empathy. Int. J. Med. Educ..

[3] Decety J., Fotopoulou A. (2015). Why empathy has a beneficial impact on others in medicine: Unifying theories. Front. Behav. Neurosci..

[4] Del Canale S., Louis D.Z., Maio V., Wang X., Rossi G., Hojat M., Gonnella J.S. (2012). The relationship between physician empathy and disease complications: An empirical study of primary care physicians and their diabetic patients in Parma, Italy. Acad. Med..

[5] Håkansson Eklund J., Holmström I.K., Ollén Lindqvist A., Sundler A.J., Hochwälder J., Marmstål Hammar L. (2019). Empathy levels among nursing students: A comparative cross-sectional study. Nurs. Open.

[6] Hojat M., Erdmann J.B., Gonnella J.S. (2013). Personality assessments and outcomes in medical education and the practice of medicine: AMEE Guide No. 79. Med. Teach..

[7] Charitou A., Fifli P., Vivilaki V.G. (2019). Is empathy an important attribute of midwives and other health professionals?: A review. Eur. J. Midwifery.

[8] Dores A.R., Martins H., Reis A.C., Carvalho I.P. (2021). Empathy and Coping in Allied Health Sciences: Gender Patterns. Healthcare.

[9] Ferri P., Rovesti S., Bonetti L., Stifani S., Panzera N., Di Lorenzo R. (2019). Evaluation of empathy among undergraduate nursing students: A three-year longitudinal study. Acta Biomed..

[10] Williams J., Stickley T. (2010). Empathy and nurse education. Nurse Educ. Today.

[11] Kim J. (2018). Factors influencing nursing students’ empathy. Korean J. Med. Educ..

[12] Maximiano-Barreto M.A., Fabrício D.M., Luchesi B.M., Chagas M.H.N. (2020). Factors associated with levels of empathy among students and professionals in the health field: A systematic review. Trends Psychiatry Psychother..

[13] Petrucci C., Gaxhja E., La Cerra C., Caponnetto V., Masotta V., Dante A., Lancia L. (2021). Empathy Levels in Albanian Health Professional Students: An Explorative Analysis Using the Jefferson Scale of Empathy. SAGE Open.

[14] Petrucci C., La Cerra C., Aloisio F., Montanari P., Lancia L. (2016). Empathy in health professional students: A comparative cross-sectional study. Nurse Educ. Today.

[15] Cunico L., Sartori R., Marognolli O., Meneghini A.M. (2012). Developing empathy in nursing students: A cohort longitudinal study. J. Clin. Nurs..

[16] Ozcan C.T., Öksüz E., Oflaz F. (2018). Improving empathy in nursing students: A comparative longitudinal study of two curricula. J. Korean Acad. Nurs..

[17] Piumatti G., Abbiati M., Baroffio A., Gerbase M.W. (2020). Empathy trajectories throughout medical school: Relationships with personality and motives for studying medicine. Adv. Health Sci. Educ. Theory Pract..

[18] Treglia E. (2020). The empathic abilities in nursing students: A longitudinal study. Clin. Ter..

[19] Winter R., Issa E., Roberts N., Norman R.I., Howick J. (2020). Assessing the effect of empathy-enhancing interventions in health education and training: A systematic review of randomised controlled trials. BMJ Open.

[20] Youssef F.F., Nunes P., Sa B., Williams S. (2014). An exploration of changes in cognitive and emotional empathy among medical students in the Caribbean. Int. J. Med. Educ..

[21] Bach R.A., Defever A.M., Chopik W.J., Konrath S.H. (2017). Geographic variation in empathy: A state-level analysis. J. Res. Pers..

[22] Berduzco-Torres N., Choquenaira-Callañaupa B., Medina P., Chihuantito-Abal L.A., Caballero S., Gallegos E., San-Martín M., Delgado Bolton R.C., Vivanco L. (2020). Factors Related to the Differential Development of Inter-Professional Collaboration Abilities in Medicine and Nursing Students. Front. Psychol..

[23] Berduzco-Torres N., Medina P., San-Martín M., Delgado Bolton R.C., Vivanco L. (2021). Non-academic factors influencing the development of empathy in undergraduate nursing students: A cross-sectional study. BMC Nurs..

[24] Bhutto Z.H., Hassan H. (2012). Empathy as a Result of Pet Ownership. Bahria J. Prof. Psychol..

[25] Faner J.M.V., Dalangin E.A.R., De Leon L.A.T.C., Francisco L.D., Sahagun Y.O., Acoba E.F. (2024). Pet attachment and prosocial attitude toward humans: The mediating role of empathy to animals. Front. Psychol..

[26] von Elm E., Altman D.G., Egger M., Pocock S.J., Gøtzsche P.C., Vandenbroucke J.P. (2008). The Strengthening the Reporting of Observational Studies in Epidemiology (STROBE) statement: Guidelines for reporting observational studies. J. Clin. Epidemiol..

[27] Albanian Pub (2015). L. No. 80. Për Arsimin E Lartë Dhe Kërkimin Shkencor Në Institucionet E Arsimit Të Lartë Në Republikën E Shqipërisë. https://arkiva.arsimi.gov.al/ligji-nr-80-2015-per-arsimin-e-larte-dhe-kerkimin-shkencor-ne-institucionet-e-arsimit-te-larte-ne-republiken-e-shqiperise-dhe-aktet-nenligjore-ne-zbatim-te-tij-2/.

[28] Althubaiti A. (2023). Sample size determination: A practical guide for health researchers. J. Gen. Fam. Med..

[29] Cohen J. (1976). Statistical Power Analysis for the Behavioral Sciences, Revised ed.

[30] Beaton D.E., Bombardier C., Guillemin F., Ferraz M.B. (2000). Guidelines for the process of cross-cultural adaptation of self-report measures. Spine (Phila Pa 1976).

[31] Draugalis J.R., Coons S.J., Plaza C.M. (2008). Best practices for survey research reports: A synopsis for authors and reviewers. Am. J. Pharm. Educ..

[32] Fincham J.E. (2008). Response rates and responsiveness for surveys, standards, and the Journal. Am. J. Pharm. Educ..

[33] Popovich D. (2025). How To Treat Missing Data In Survey Research. J. Mark. Theory Pract..

[34] Caponnetto V., Dante A., Masotta V., La Cerra C., Petrucci C., Alfes C.M., Lancia L. (2021). Examining nursing student academic outcomes: A forty-year systematic review and meta-analysis. Nurse Educ. Today.

[35] Chen D.C., Kirshenbaum D.S., Yan J., Kirshenbaum E., Aseltine R.H. (2012). Characterizing changes in student empathy throughout medical school. Med. Teach..

[36] Bosméan L., Chaffanjon P., Bellier A. (2022). Impact of physician-patient relationship training on medical students’ interpersonal skills during simulated medical consultations: A cross-sectional study. BMC Med. Educ..

[37] Chen C.H., Wang S.J., Yeh W.Y., Wu C.L., Wang Y.A., Chen C.F., Yang Y.Y., Huang W.J., Chan K.Y., Lai C.W. (2022). Evaluating Teaching Effectiveness of Medical Humanities in an Integrated Clerkship Program by a Novel Prospective Propensity Score Matching Framework. Int. J. Environ. Res. Public Health.

[38] Crawford D.R. (2021). Compassion and Empathy in Basic Medical Science Teaching: A Suggested Model. Cureus.

[39] Adamson K., Sengsavang S., Charise A., Wall S., Kinross L., Balkaran M. (2018). Narrative training as a method to promote nursing empathy within a pediatric rehabilitation setting. J. Pediatr. Nurs..

[40] Lwow M., Canetti L., Muszkat M. (2020). Gender differences in the effect of medical humanities program on medical students’ empathy: A prospective longitudinal study. BMC Med. Educ..

[41] Roberts M.L., Kaur T. (2023). Effect of Storytelling and Empathy Training to Support Affective Learning in Undergraduate Nursing Students. Nurse Educ..

[42] Seney V. (2023). It Could Have Been Me: Nursing Students’ Self-reflection to Enhance Empathy in Caring for Those with Substance Use Disorder. Nurse Educ..

[43] Varagona L., Myers R., Wilson A. (2021). Weekly Email Reminders Increase Nursing Students’ Use of Empathic Communication: A Randomized Controlled Trial. Nurse Educ..

[44] Barisone M., Ghirotto L., Busca E., Diaz Crescitelli M.E., Casalino M., Chilin G., Milani S., Sanvito P., Suardi B., Follenzi A. (2022). Nursing students’ clinical placement experiences during the COVID-19 pandemic: A phenomenological study. Nurse Educ. Pract..

